# Ciprofloxacin-Resistant *Pseudomonas aeruginosa* Lung Abscess Complicating COVID-19 Treated with the Novel Oral Fluoroquinolone Delafloxacin

**DOI:** 10.1155/2022/1008330

**Published:** 2022-02-16

**Authors:** Jürgen Panholzer, Matthias Neuboeck, Guangyu Shao, Sven Heldt, Markus Winkler, Paul Greiner, Norbert Fritsch, Bernd Lamprecht, Helmut Salzer

**Affiliations:** ^1^Department of Pulmonology, Kepler University Hospital, Linz, Austria; ^2^Departments of Pathology and Microbiology, Kepler University Hospital, Linz, Austria; ^3^Department of Internal Medicine, Freistadt Hospital, Freistadt, Austria

## Abstract

**Purpose:**

We report the development of a lung abscess caused by a ciprofloxacin-resistant Pseudomonas aeruginosa in a patient with COVID-19 on long-term corticosteroid therapy. Successful antimicrobial treatment included the novel oral fluoroquinolone delafloxacin suggesting an oral administration option for ciprofloxacin-resistant Pseudomonas aeruginosa lung abscess. *Case Presentation.* An 86-year-old male was admitted to the hospital with fever, dry cough, and fatigue. PCR testing from a nasopharyngeal swab confirmed SARS-CoV-2 infection. An initial CT scan of the chest showed COVID-19 typical peripheral ground-glass opacities of both lungs. The patient required supplemental oxygen, and anti-inflammatory treatment with corticosteroids was initiated. After four weeks of corticosteroid therapy, the follow-up CT scan of the chest suddenly showed a new cavernous formation in the right lower lung lobe. The patient's condition deteriorated requiring high-flow oxygen support. Consequently, the patient was transferred to the intensive care unit. Empiric therapy with intravenous piperacillin/tazobactam was started. Mycobacterial and fungal infections were excluded, while all sputum samples revealed cultural growth of *P. aeruginosa.* Antimicrobial susceptibility testing showed resistance to meropenem, imipenem, ciprofloxacin, gentamicin, and tobramycin. After two weeks of treatment with intravenous piperacillin/tazobactam, the clinical condition improved significantly, and supplemental oxygen could be stopped. Subsequently antimicrobial treatment was switched to oral delafloxacin facilitating an outpatient management.

**Conclusion:**

Our case demonstrates that long-term corticosteroid administration in severe COVID-19 can result in severe bacterial coinfections including *P. aeruginosa* lung abscess. To our knowledge, this is the first reported case of a *P. aeruginosa* lung abscess whose successful therapy included oral delafloxacin. This is important because real-life data for the novel drug delafloxacin are scarce, and fluoroquinolones are the only reliable oral treatment option for *P. aeruginosa* infection. Even more importantly, our case suggests an oral therapy option for *P. aeruginosa* lung abscess in case of resistance to ciprofloxacin, the most widely used fluoroquinolone in *P. aeruginosa* infection.

## 1. Introduction

In December 2019, the novel severe acute respiratory syndrome coronavirus 2 (SARS-CoV-2) emerged causing coronavirus disease 2019 (COVID-19), which rapidly became a global health emergency. The clinical spectrum ranges from asymptomatic to COVID-19 typical symptoms including high fever, dry cough, shortness of breath, fatigue, myalgia, headache, abdominal discomfort, or loss of taste and smell [1]. Risk factors for a severe course of diseases or death are amongst others high age (>60 years), male gender, hypertension, COPD, and diabetes [2]. Diagnosis is based on PCR or antigen testing from a naso- or oropharyngeal swab. Laboratory findings often include leucopenia and thrombocytopenia, and the radiological pattern typically shows peripheral ground-glass opacities of both lungs [3]. Several agents are approved or are under investigation including antivirals, antibodies, immunomodulators, and corticosteroids. The latter is established as standard of care in severe and critically ill COVID-19 patients since a multicenter randomized controlled trial has shown significant reducing duration of mechanical ventilation mortality in moderate-to-severe ARDS [4, 5].

In about 7% of patients, COVID-19 is complicated by secondary coinfections. While in influenza, bacterial coinfections emerge in up to 65% of patients, COVID-19 seems to have a rather low coinfection rate [6, 7]. This is surprising as COVID-19 is associated with several immunosuppressive factors such as high age, diabetes, and corticosteroid therapy [3, 4]. If bacterial coinfection appears, it is often associated with a severe course of COVID-19 and with a poorer outcome [8].

Bacterial coinfection may be caused by *Pseudomonas aeruginosa*, which is usually treated with piperacillin/tazobactam, ceftazidim, carbapenems, or ciprofloxacin; however, in case of resistance, treatment options are very limited. Delafloxacin (Melinta Therapeutics, USA, Connecticut) is a novel fluoroquinolone for the respiratory tract and skin infections addressing *P. aeruginosa* and other multidrug-resistant pathogens such as methicillin-resistant *Staphylococcus aureus* (MRSA). Compared to other fluoroquinolones, delafloxacin is also available in an oral formulation, while most other antibacterial alternatives for *P. aeruginosa* are only administrable intravenously [9, 10].

We report the development of a lung abscess caused by a ciprofloxacin-resistant *Pseudomonas aeruginosa* in a patient with COVID-19 on long-term corticosteroid therapy. Successful antimicrobial treatment included the novel oral fluoroquinolone delafloxacin.

## 2. Case Presentation

**Figure 1:**
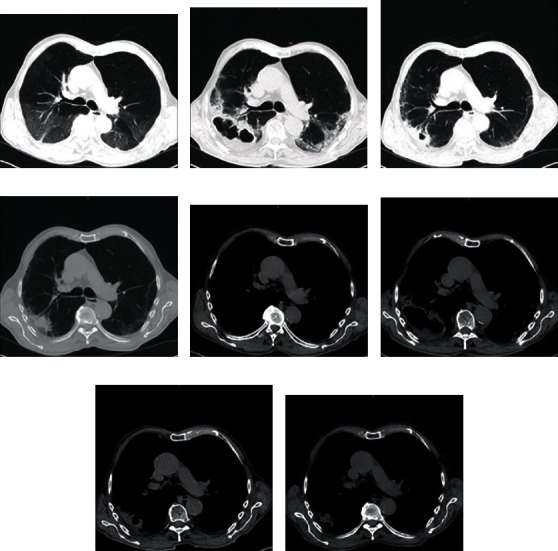
Axial chest CT scans showing peripheral ground-glass opacities of both lungs at hospital admission (a, e), a large cavitary lesion at the right inferior lung lobe at the end of corticosteroid treatment (b, f), a declining cavitary lesion at the end of antibacterial therapy (c, g), and a small consolidation at two-month follow-up (d, f).

**Figure 2:**
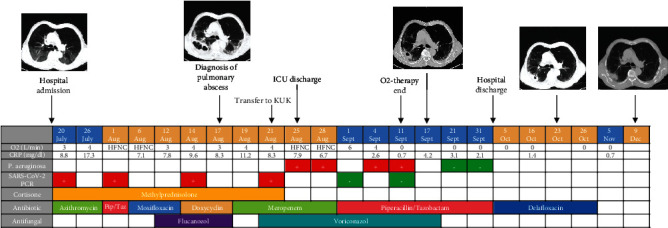
Timeline showing the key parameters and therapies. KUK = Kepler University Hospital; ICU = intensive care unit; Aug = August; Sept = September; Oct = October; Nov = November; Dec = December; HFNC = high-flow nasal cannula oxygen; CRP = C-reactive protein; SARS-CoV-2 = severe acute respiratory syndrome coronavirus type 2; PCR = polymerase chain reaction; Pip/Taz = piperacillin/tazobactam.

An 86-year-old man was admitted to the hospital on July 20^th^, 2020, with fever, dry cough, and fatigue. Medical history revealed an arterial hypertension, recurrent pulmonary embolism in 1993 and in 2007, and a prostate carcinoma. The last days before admission, he took ciprofloxacin 500 mg two times daily (bid) because of a recent urinary tract infection. His vital signs were normal. Auscultation revealed fine crackles in both basal lung lobes. Cardiac, abdominal, and neurological examination was unremarkable. Laboratory tests showed a leucopenia of 4.07 G/L (3.9-8.8 G/L), a decreased lymphocyte count of 0.3 G/L (1.0-4.8 G/L), and an elevated C-reactive protein (CrP) level of 7.73 mg/dL (0-0.5 mg/dL), while the procalcitonin level was normal. Polymerase chain reaction (PCR, Roche, Basel, Switzerland) from a nasopharyngeal swab confirmed SARS-CoV-2 infection. A computed tomography (CT) scan of the chest showed peripheral distributed ground-glass opacities in all lung lobes (Figures 1(a) and 1(e)). Treatment with methylprednisolone 32 mg once a day (od) and empiric antimicrobial therapy with azithromycin 500 mg was initiated. Blood cultures showed no bacterial growth. The patient's condition deteriorated, and antimicrobial therapy was switched several times (Figure 2). A follow-up chest CT scan on August 17^th^, 2020, showed a new cavernous formation in the right lower lung lobe (Figures 1(b) and 1(f)). Due to the radiological presentation, a fungal infection was suspected, and fluconazole 200 mg was started.

As the patient's condition further deteriorated and the oxygen demand increased, the patient was transferred to the local university hospital. Antibacterial treatment with meropenem 2 g three times a day (tid) i.v. and corticosteroid therapy was continued, while antifungal treatment was switched to voriconazole 200 mg bid intravenously after a loading dose on day 1.

Again, blood samples were negative for bacterial growth. The galactomannan test (IMMY, Oklahoma, USA) from serum was negative. Microscopy for acid-fast bacilli as well as PCR for *M. tuberculosis*-complex from sputum was negative. Sputum cultures did not show fungal growth but revealed growth of *P. aeruginosa* on August 25^th^, 2020. Moreover, multiple skin swabs (throat, groin, and perianal) were also positive for *P. aeruginosa*. Bronchial secretion obtained during bronchoscopy from the lower right lung lobe also revealed cultural growth of *P. aeruginosa*, while the galactomannan test and fluorescence microscopy for *Pneumocystis jirovecii* from bronchoalveolar lavage were negative.

Antimicrobial susceptibility testing (Thermo Fisher Scientific, Massachusetts, USA) of *P. aeruginosa* showed resistance to meropenem, imipenem, ciprofloxacin, gentamicin, and tobramycin. Antibacterial treatment was switched on August 28^th^, 2020, to piperacillin/tazobactam 4.5 g tid i.v., while antifungal treatment with voriconazole was stopped. This targeted treatment regime resulted in rapid clinical and radiological improvement enabling to stop oxygen supplementation therapy on September 11^th^, 2020. Bacterial sputum conversion was achieved on September 21^st^, 2020. The first negative SARS-CoV-2 PCR was documented on September 1^st^, 2020.

Since our patient did not require additional oxygen anymore, the only reason to be treated on the ward was the i.v. administration of piperacillin/tazobactam for the treatment of the ciprofloxacin-resistant *P. aeruginosa* lung abscess. A service for an outpatient parenteral therapy (OPAT) is not available in Austria so far. Therefore, the treatment strategy was changed to the novel fluoroquinolone delafloxacin characterized by a sufficient bioavailability when taken orally [9, 10].

We discharged the patient at the beginning of October with oral delafloxacin 450 mg bid. Antibacterial treatment was well tolerated, and the patient showed continuous radiological improvement. Delafloxacin treatment was stopped by the end of October 2020.

## 3. Discussion

We report the development of a lung abscess caused by a ciprofloxacin-resistant *Pseudomonas aeruginosa* in a patient with COVID-19 on long-term corticosteroid therapy. Successful antimicrobial treatment included the novel oral fluoroquinolone delafloxacin.

Current literature indicates an overall bacterial coinfection rate of 7 to 14% in COVID-19 [7]. These rates are lower compared to the frequency of coinfections in influenza, where most studies report rates ranging from 11 to 35% [6]. Hospital-associated coinfections occur in about 12% of COVID-19 patients, while community-associated coinfections are less frequently seen with around 6% of cases [11]. Surprisingly, there are several studies showing high usage of antibacterial treatment in COVID-19, while the risk of coinfections seems to be rather low. A recent study by Kubin et al. reported 67% of patients receiving at least 1 dose of antibacterial therapy [11, 12]. However, when bacterial coinfection does appear, it is associated with both increased severity of COVID-19 and poorer outcome [8]. Antimicrobial stewardship principles should not be neglected due to concerns of availability for the global supply chain preventing therapy for those who need it, the increased workload with parenteral administration, and long-term complications associated with antibacterial overuse including development of resistance [13].

We suggest that our patient had several risk factors for developing a bacterial lung abscess including severe illness requiring ICU stay, high age, multiple antibacterial therapies, and long-term corticosteroid treatment. Our patient was on corticosteroid treatment for almost a month, while most guidelines including the Infectious Disease Society of America (IDSA) guideline recommend a total of 10 days for patients with severe COVID-19. Given the hyperinflammatory state in severe COVID-19 patients, immunomodulatory approaches including steroids, but also others such as tocilizumab, are continuously under investigation [4]. A multicenter randomized controlled trial investigating the use of dexamethasone demonstrated a reduction of the 28-day mortality and a reduction of ventilator-associated days [5]. Furthermore, there are studies showing increased coinfection and mortality rates in viral pneumonia other than COVID-19 treated with corticosteroids [14, 15].

Cavitation can also be associated with fungal infections such as pulmonary aspergillosis [16]. The risk for COVID-19-associated pulmonary aspergillosis (CAPA) in critically ill COVID-19 patients is estimated about 5-30% and is associated with corticosteroid therapy [17]. Recent studies showed an increased mortality in COVID-19 complicated by a CAPA compared with patients without CAPA (36% versus 9.5%). Serum and bronchoalveolar lavage galactomannan testing yields high sensitivity and specificity for diagnosing CAPA offering early antifungal treatment [18].

In our case, two weeks of intravenous piperacillin/tazobactam treatment improved the clinical condition significantly and supplemental oxygen could be stopped. Due to structural issues in Austria, it was not possible to organize OPAT. Delafloxacin is a novel fluoroquinolone, which is available in an oral formulation [9, 10]. Some experts indicate its low bioavailability, which may have the risk to provoke resistance. In fact, bioavailability for the oral formulation may be below 60%, while 35-50% of its oral administered dose is eliminated unchanged via feces. On the other hand, minimal inhibitory concentrations are three- to five-fold lower for gram-positive strains and four- to seven-fold lower for gram-negative strains compared to other fluoroquinolones. Additionally, in vitro tests showed similar resistance rates to moxifloxacin and lower rates compared to levofloxacin [19]. Furthermore, delafloxacin lacks clinical relevant Qt-prolongation and phototoxicity compared to other fluoroquinolones [9, 20]. An oral treatment alternative for *P. aeruginosa* could reduce the duration of hospitalization, complications, and costs compared to i.v. drugs.

Limitations of our case report are that we were not able to perform antimicrobial susceptibility testing for delafloxacin, because the patient already achieved sputum conversion at the time, we considered delafloxacin the first time, and unfortunately no *P. aeruginosa* isolates were stored. However, we are convinced that delafloxacin was susceptible since we saw that the radiological pattern further improved under the treatment.

In conclusion, we suggest an increased risk of bacterial coinfections including lung abscess in COVID-19 patients on long-term corticosteroid therapy. Therefore, the decision to exceed the recommended duration for corticosteroid administration in severe COVID-19 patients should be critically evaluated based on individual circumstances. To our knowledge, this is the first reported case of a *P. aeruginosa* lung abscess whose successful therapy included oral delafloxacin. This is important because real-life data for the novel drug delafloxacin are scarce, and fluoroquinolones are the only reliable oral treatment option for *P. aeruginosa* infection. Even more importantly, our case suggests an oral therapy option for *P. aeruginosa* lung abscess in case of resistance to ciprofloxacin, the most widely used fluoroquinolone in *P. aeruginosa* infection. Oral treatment options are important because they enable outpatient care and reduce duration of hospitalization, complications, and costs compared to i.v. drugs. The lack of clinically relevant Qt-prolongation and phototoxicity compared to other fluoroquinolones as reported by the previous literature makes the use of delafloxacin even more intriguing.

## Data Availability

Data will be provided by contacting Juergen Panholzer or Helmut Salzer via the abovementioned email addresses.
